# Photoprotective Effects of Epigallocatechin Gallate on Ultraviolet-Induced Zebrafish and Human Skin Fibroblasts Cells

**DOI:** 10.1155/2024/7887678

**Published:** 2024-01-24

**Authors:** Jie Zhang, Yahui Xu, Xiyu Ruan, Ting Zhang, Minghui Zi, Qiao Zhang

**Affiliations:** Yunnan Provincial Key Laboratory of Public Health and Biosafety and School of Public Health, Kunming Medical University, Kunming 650500, China

## Abstract

**Background:**

The long-term exposure to ultraviolet radiation (UVR) raises oxidative stress and chronic inflammation levels, which in turn has a series of deleterious effects on skin health, such as sunburn, photoaging, and skin cancer. Hence, our study was determined to investigate the effects and mechanisms of epigallocatechin gallate (EGCG) in zebrafish and human skin fibroblasts (HSF) cells to alleviate ultraviolet-induced photoaging.

**Methods:**

The 4 days postfertilization (dpf) zebrafish larvae and HSF cells were treated with 10 J/cm^2^ UVA + 30 mJ/cm^2^ UVB, or 25, or 50 *μ*M EGCG for 72 hr. The indicators involving in oxidative stress, inflammatory, and photoaging were measured by the kits, ELISA Kits and western blot methods.

**Results:**

EGCGs protect against UVR-induced skin damage in zebrafish and HSF cells. EGCG markedly decreased the reactive oxygen species (ROS), malondialdehyde, 8-OHdG levels, increased superoxide dismutase (SOD) activity, and significantly inhibited inflammatory factors levels including tumor necrosis factor-*α* (TNF-*α*), interleukin-1*α* (IL-1*α*), interleukin-6 (IL-6) in zebrafish, and HSF cells irradiated with UVR. We found that EGCG could reduce UVR-induced p38 mitogen-activated protein kinase (p38 MAPK) phosphorylation and effectively inhibited the activity of the transcriptional factor nuclear factor-*κ*B (NF-*κ*B), thereby reducing the protein-1 (AP-1), TNF-*α*, IL-1*α*, IL-6, and matrix metalloproteinase-1 (MMP-1) expressions, which are critical mediators of skin aging cascade causing the photoaging.

**Conclusion:**

These results validate that EGCG for protection of photoaging in zebrafish and HSF cells induced by UVR, which is closely related to the regulation of p38 MAPK/NF-*κ*B, AP-1 signaling pathway which relieve oxidative stress, inflammation, and collagen degradation.

## 1. Introduction

Ultraviolet radiation (UVR) is a major environmental source of damage behind the skin damage process, like solar dermatitis, edema, erythema, photoaging, and skin-related diseases, especially photoaging [1]. According to wave length, it is classified as ultraviolet A (315–400 nm), B (280–315 nm), and C (100–280 nm). The UVRs that can reach the earth are UVA (95%) and UVB (5%) [2]. UVA has strong penetration ability, catalyzes the degradation of elastin and collagen, accelerates skin aging, emerge in wrinkles, melasma, actinic keratoses, and texture changes, which is the main factor causing photoaging in human. UVB has high energy and relatively weak penetration ability, however, it can also penetrate this skin layer to reach the dermis and affect the physiological functions of fibroblasts [3].

The long-term exposure to UVR, which is easier to cause photochronical generation of reactive oxygen species (ROS), that activates cell surface growth factors, cytokine receptors, and nicotinamide adenine dinucleotide phosphate (NADPH) oxidase [4]. Additionally, the accumulations of ROS also further to result in chronic inflammatory factors, collagen degradation, and an increase matrix metalloproteinase (MMPs) activity. In particular, nuclear transcription factors, activator protein 1 (AP-1), and nuclear factor-*κ*B (NF-*κ*B), involved in transcription of genes for matrix-degrading enzymes and proinflammatory cytokines, respectively, are activated, which results in the abnormal morphological changes, and the structural and functional alterations shown in the skin [5, 6]. The paper reported that UVB-induced melanogenesis by antagonizing cAMP/PKA and ROS/MAPK signaling pathways [7]. Moreover, increased levels of ROS production are responsible for the oxidative stress, leading to macromolecules and DNA damage, and further initiating the oxidative-stress sensitive transcription factors (Nrf2, NF-*κ*B, AP-1, and p53) and protein kinases (MSK, ERK, JNK, p38 MAPK, p90RSK2, and CaMKs) involved in inflammation, immune suppression, apoptosis, extracellular matrix (ECM) remodeling, photoaging, senescence, and photo-carcinogenesis [8]. Conversely, mitigating UVR-induced oxidative stress is one of the effective approaches to prevent photoaging and skin cancer. It is possible that free radical scavengers can prevent UVR-induced skin damage by suppressing the induction of p38 mitogen-activated protein kinase (p38 MAPK) phosphorylation and effectively inhibiting the activity of the transcriptional NF-*κ*B, thereby reducing AP-1, MMPs, tumor necrosis factor-*α* (TNF-*α*) expressions.

Catechins, as the primary components of green tea, include epigallocatechin gallate (EGCG), epigallocatechin (EGC), epicatechin gallate (ECG), and epicatechin (EC). Among these, EGCG is the major component of green tea polyphenols, which have been studied in many beneficial effects, such as anti-inflammatory, antioxidative, antimicrobial, and anticancer effects [9]. The study reported that EGCG could be a potential strategy in preventing human skin fibroblasts (HSF) photoaging induced by UVA radiation, is closely related to its powerful antioxidant effects to regulate the expression of related genes, like tissue inhibitor of metalloproteinase-1 (TIMP-1), p66 [10]. EGCG counters UVB-mediated phototoxicity via inhibition of the epidermal growth factor receptor in keratinocytes [11, 12]. Recently, the studies have primarily focused on UVA or UVB-irradiated skin damage and photoaging in cells or mice. The underlying mechanisms by which EGCG on UVR (UVA + UVB) in human skin caused by alleviating oxidative stress and inflammation remain unclear. Moreover, the zebrafish (*Danio rerio*) is an emerging model organism that serves as vertebrate development, biomedical research, drug development, and clinical therapy. This is main due to the high level of genome structure shared between zebrafish and humans, easy maintenance, year-round spawning, high fecundity (300–600 by single female at one time), undergo rapid external development, optical transparency of early stages, and common molecular pathways can be studied [13, 14]. Therefore, our study investigated the protective effects and mechanisms of EGCG in the development of oxidative stress and inflammation caused by UVR-induced photoaging in zebrafish and HSF cells.

## 2. Materials and Methods

### 2.1. Materials

Dulbecco's modifed eagle medium (DMEM) was obtained from Gibco (Grand Island, NY); fetal bovine serum (FBS) was obtained from TransGen Biotech (TransGen Biotech Co., Ltd., Beijing, China); EGCG and dimethyl sulfoxide (DMSO) for cell experiments were obtained from Sigma–Aldrich (St. Louis, MO, USA)); 3-(4,5-dimethylthiazol-2-yl)−2,5-diphenyl−2 H-tetrazolium bromide (MTT) for cytotoxicity was purchased from MP Biomedicals (Biofroxx, Shanghai, China); GAPDH was obtained from Santa Cruz Biotechnology (Santa Cruz, CA, USA). Total collagen assay was purchased from Cell Signaling Technology (Danvers, MA, USA).

### 2.2. Effects of EGCG on Zebrafish Growth

Embryos of AB wild-type zebrafish developed at 3 days postfertilization (dpf) were collected and randomly divided into 80 zebrafish per experimental group. The 4 dpf zebrafish larval were treated with 0, 6.25, 12.5, 25,50, 100 *μ*M EGCG for 24, 48, and 72 hr, and exposed to 10 J/cm^2^ UVA + 30 mJ/cm^2^ UVB generated by an ultraviolet light therapy instrument (HT-H150, Shenzhen, China) and measured intensity by a radiometer (TS280MAX, Suderui Biotechnology, ShenZhen, China) [15]. For UVR exposure, each group was exposed 15 min every day for 24, 48, and 72 hr. After UVR exposure, all zebrafish larval were cultivated in culture dishes and their survival rates were counted at 5, 6, and 7 dpf.

### 2.3. Repairing Effect of EGCG on Caudal Fin Damage in UVR-Induced Zebrafish Larvae

The 3 dpf zebrafish larvae were collected and randomly divided into 80 zebrafish per experimental group. Zebrafish larvae were treated with UVR exposure and/or 25 *μ*M EGCG at 4 dpf for 72 hr. The zebrafish larvae were cultivated in the culture dishes and culture medium was changed daily throughout the experiment at 28°C until 7 dpf. Ten zebrafish larvae were randomly chosen from each experimental group to observe their tails under microscope (Nikon 800SMZ−800 N, Tokyo, Japan). Images were acquired and analyzed using the Image View software to calculate the caudal fin area of the zebrafish larvae, which was used to assess the repair effect of EGCG on UVR-induced skin damage in zebrafish.

### 2.4. Cell Culture and Treatment

HSF cell line was obtained from the Chinese Academy of Science (Shanghai, China). The cells were grown in DMEM supplemented with 10% fetal bovine serum and 1% antibiotic/antimycotic at 37°C in an atmosphere containing 95% air and 5% CO_2_. The HSF cells were treated with UVR and 50 *μ*M EGCG or without UVR or EGCG (control) for 72 hr.

### 2.5. Cell Viability Analysis

The cell viability was evaluated using 3-(4, 5-dimethylthiazol−2-yl)−2, 5-diphenyltetrazolium bromide (MTT) assay. In brief, HSF cells were cultured overnight in a 96-well plate at a density of 1 × 10^5^ cells/well. After being treated with 0–800 *μ*mol/L EGCG for 24, 48, and 72 hr, 100 *μ*L of phosphate buffered saline (PBS) buffer for washing. Then, 20 *μ*L of MTT solution (5 mg/mL in PBS as the stock solution) was added to each culture well, and the plate was incubated at 37°C for 4 hr in the dark. The medium was replaced by 150 *μ*L (per well) dimethyl sulfoxide (DMSO) and left for 10 min at room temperature. The percentage of viable cells was assessed by a plate reader at 570 nm [16].

### 2.6. Senescence-Associated *β*-Galactosidase Staining

The zebrafish and HSF cells senescence was assessed by *β*-galactosidase (Beyotime Biotechnology, Shanghai, China) staining, according to the manufacturers' instructions.

### 2.7. The Assays of Oxidative Stress and Inflammatory Indicators

ROS was assayed using commercial kits from Nanjing Jiancheng Institute of Biological Engineering (Nanjing, China) using the dichloro-dihydrofluorescein diacetate (DCFH-DA) method, based on the ROS dependent oxidation of DCFH to the highly fluorescent DCF. HSF cells were seeded at a density of 1 × 10^4^ cells/well. About, 10 *μ*M DCFH-DA was added to each well after treatment for 72 hr, and the plate was incubated at 37°C in the dark for 30 min. Subsequently, single-cell suspension was collected and removed supernatant, the cell precipitate was washed 1–2 times with PBS to fully removed DCFH-DA that has not entered the cell. The fluorescence was determined immediately at 488-nm excitation wavelength and 525-nm emission wavelength by fluorescence microscope (Nikon ECLIPSE Ts2R) and the FITC-A value using a flow cytometer (BD LSR Fortessa) within 20 s for each measurement [17]. SOD activity (Nanjing Jiancheng Institute of Biological Engineering, Nanjing, China), MDA (Nanjing Jiancheng Institute of Biological Engineering, Nanjing, China), and 8-OHdG (JINGMEI, Jiangsu, China) were measured according to directions of commercial kits. IL−1*α* (Jinhengnuo Biotechnology, Hangzhou, China), TNF-*α*, and IL-6 (Ray biotech, Atlanta, American) were determined by an enzyme-linked immunosorbent assay (ELISA) using commercial kits in the zebrafish according to the manufacturers' instructions. TNF-*α*, IL-1*α*, and IL-6 were determined by ELISA using commercial kits (ABclonal Biotechnology, Wuhan, China) in HSF cells according to the manufacturers' instructions.

### 2.8. Western Blot

The zebrafish larvae and HSF cells were extracted and centrifuged in EP tube at 12,000 rpm for 20 min, with supplement of protease inhibitors (Roche, Indianapolis, IN). The protein concentration was determined by BCA Protein Assay Kit (Beyotime Biotechnology, Shanghai, China). About, 30–40 *μ*g of protein was loaded onto 10% or 12% SDS-PAGE gel and was transferred to PVDF membranes (Millipore, Billerica, MA). The proteins, including *β*-actin (ABclonal Biotechnology, Wuhan, China, MW42), phospho-p38 MAPK, p38 MAPK (ABclonal Biotechnology, Wuhan, China, MW41), *c*-jun (Cell signaling Technology, MW43), phospho-*c*-jun (Cell signaling Technology, MW48), NF-*κ*B (ABclonal Biotechnology, Wuhan, China, MW65), MMP1 (ABclonal Biotechnology, Wuhan, China, MW62), IL-6 (Affinity Biosciences, Nanjing, China, MW24), IL-1*α* (ABclonal Biotechnology, Wuhan, China, MW20), and TNF-*α* (Affinity Biosciences, Nanjing, China, MW17) proteins were incubated overnight at 4°C. The next day, anti-rabbit alkaline phosphatase-conjugated antibody (KPL, Los Angeles, USA) was used as a secondary antibody for 1 hr at 30°C. Protein signals were detected via ECL method [18, 19].

### 2.9. Statistical Analysis

All statistical analyses were used by the Statistical Package of Social Sciences (SPSS) 18.0 software and conducted by the one-way ANOVA, pairwise comparisons using the Dunnett's test, results were presented as mean ± standard deviation and *P* < 0.05 was considered to be statistically significant.

## 3. Results

### 3.1. Toxicity Effects of EGCG on Zebrafish and HSF Cells

**Figure 1:**
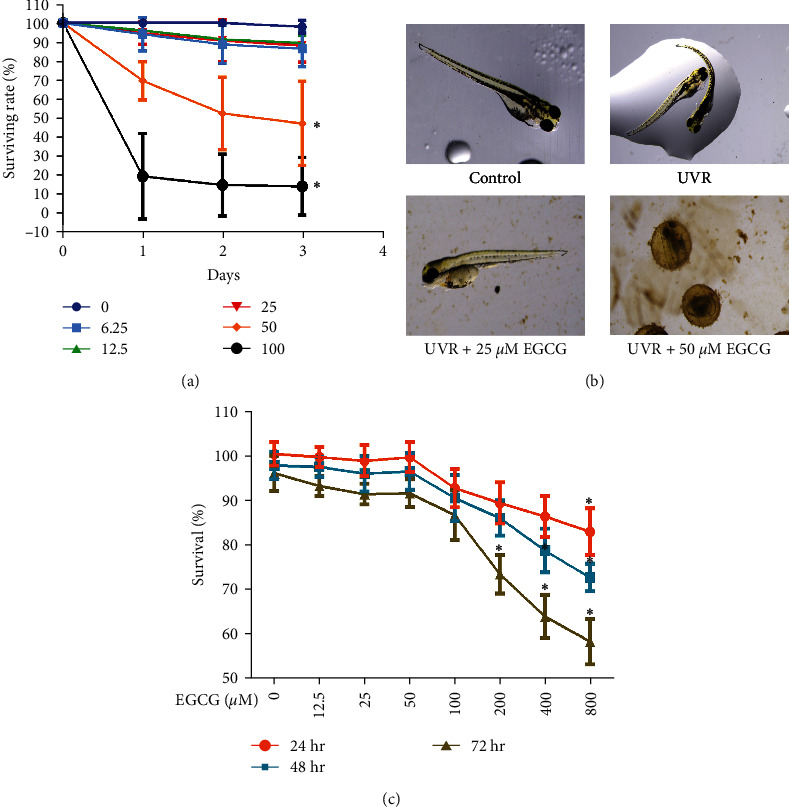
The toxicity effects of EGCG in zebrafish and HSF cells. The effects of EGCG (0, 6.25, 12.5, 25, 50, 100 *μ*M) on zebrafish larvae for 72 hr (a, b). The effects of EGCG (0, 12.5, 25, 50, 100, 200, 400, 800 *μ*M) for 72 hr with MTT assay (c). The experiments were repeated three times. Data are presented as means ± SD (*n* = 3).  ^*∗*^*P* < 0.05, compared with control group.

Zebrafish larvae were exposed to 0–100 *μ*M of EGCG for 72 hr to evaluate the survival percentage (Figure 1(a)). HSF cells were treated with 0–800 *μ*M of EGCG for 72 hr to assess cell viability by MTT assay (Figure 1(b)). The toxicity was observed when the concentration of EGCG reached 50 *μ*M in zebrafish larvae, and 200 *μ*M in HSF cells for 72 hr. Thus, the concentration of 25 *μ*M EGCG was selected in zebrafish larvae and 50 *μ*M for HSF cells for 72 hr in this study.

### 3.2. EGCG Protect against UVR-Induced Skin Damage and Skin Aging in Zebrafish and HSF Cells

**Figure 2:**
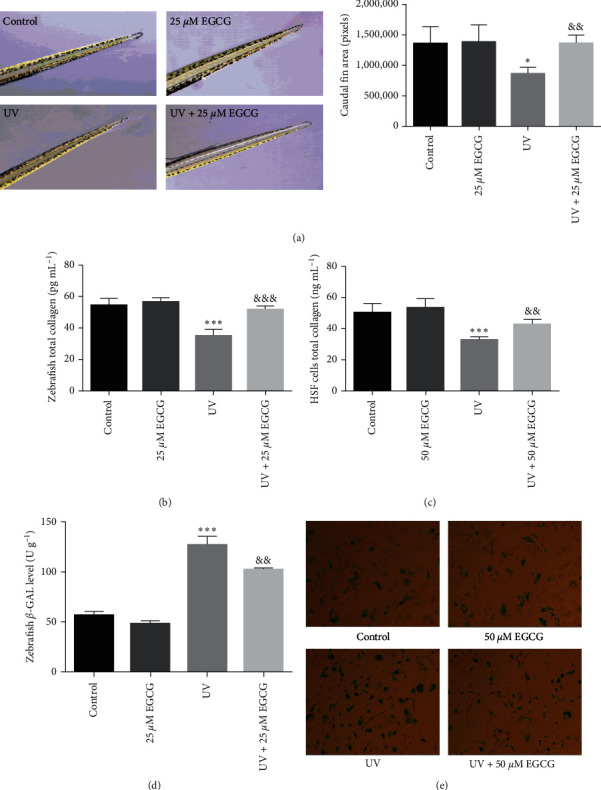
The effects of EGCG on caudal fin damage, total collagen and SA *β*-gal level. The effects of EGCG on caudal fin damage (a) total collagen (b, c) and SA *β*-gal level (d, e) in UVR-induced zebrafish (25 *μ*M) and HSF cells (50 *μ*M) for 72 hr. The experiments were repeated three times. Data are presented as means ± SD (*n* = 3).  ^*∗*^*P* < 0.05,  ^*∗∗*^*P* < 0.01, and  ^*∗∗∗*^*P* < 0.001 compared with control group. ^&^*P* < 0.05, ^&&^*P* < 0.01, and ^&&&^*P* < 0.001 compared with UVR group.

The repair effect of EGCG on caudal fin damage in zebrafish larvae exposed to UVR in Figure 2(a). The caudal fin of zebrafish larvae exhibited a rough and crinkled phenotype after exposure to UVR under microscope. The repair of zebrafish caudal fin damage was 59% with 25 *μ*M EGCG, suggesting that EGCG treatment markedly repaired UVR-induced skin damage in zebrafish larvae. Moreover, the EGCG-treated group displayed much less collagen damage that results in skin aging than the UVR group and also prevented the reduction of collagen synthesis (Figures 2(b) and 2(c)). Meanwhile, the number of cells positive for senescence-associated beta-galactosidase (SA *β*-gal) marked decrease in the EGCG group (Figures 2(d) and 2(e)). Collectively, these results verify that EGCG prevent UVR-induced skin damage and photoaging in zebrafish and HSF cells.

### 3.3. EGCG Inhibited UVR-Induced Oxidative Stress and Inflammation in Zebrafish and HSF Cells

**Figure 3:**
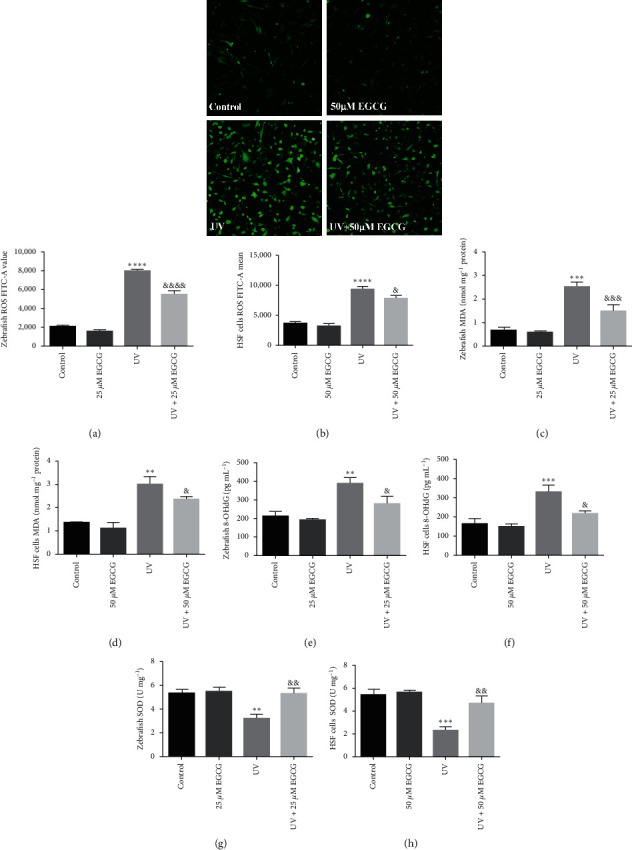
The effects of EGCG on oxidative stress factors levels in zebrafish larvae and HSF cells. The effects of EGCG on oxidative stress factors (a–h) in UVR-induced zebrafish larvae (25 *μ*M EGCG) and HSF cells (50 *μ*M EGCG) for 72 hr. The experiments were repeated three times. Data are presented as means ± SD (*n* = 3).  ^*∗*^*P* < 0.05,  ^*∗∗*^*P* < 0.01,  ^*∗∗∗*^*P* < 0.001, and  ^*∗∗∗∗*^*P* < 0.0001 compared with control group. ^&^*P* < 0.05, ^&&^*P* < 0.01, ^&&&^*P* < 0.001, and ^&&&&^*P* < 0.0001 compared with UVR group.

**Figure 4:**
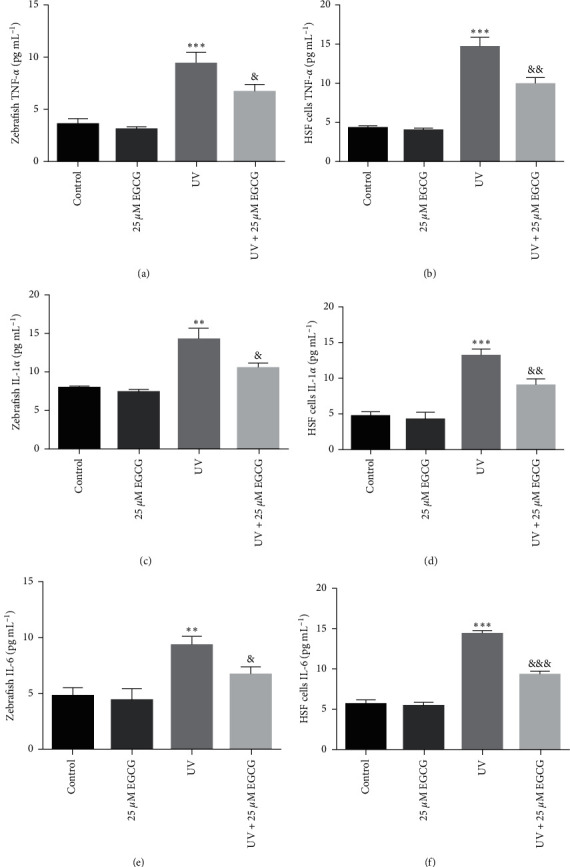
The effects of EGCG on inflammation factors levels in zebrafish larvae and HSF cells. The effects of EGCG on inflammation factors level (a−f) in UVR-induced zebrafish (25 *μ*M EGCG) and HSF cells (50 *μ*M EGCG) for 72 hr. The experiments were repeated three times. Data are presented as means ± SD (*n* = 3).  ^*∗*^*P* < 0.05,  ^*∗∗*^*P* < 0.01, and  ^*∗∗∗*^*P* < 0.001 compared with control group. ^&^*P* < 0.05, ^&&^*P* < 0.01, and ^&&&^*P* < 0.001 compared with UVR group.

The oxidative stress and inflammation factors significantly increased after treatment with UVR (*P* < 0.05). EGCG treatment markedly reduced levels of ROS (Figures 3(a) and 3(b)), MDA (Figures 3(c) and 3(d)), 8-OHdG (Figures 3(e) and 3(f)), and upregulated the antioxidant enzymes, SOD (Figures 3(g) and 3(h)) activity. Meanwhile, EGCG also obviously downregulated the UVR-induced intracellular levels of TNF-*α* (Figures 4(a) and 4(b)), IL-1*α* (Figures 4(c) and 4(d)), and IL-6 (Figures 4(e) and 4(f)).

### 3.4. EGCG Modulated on the Key Proteins Involved in Photoaging in Zebrafish and HSF Cells

**Figure 5:**
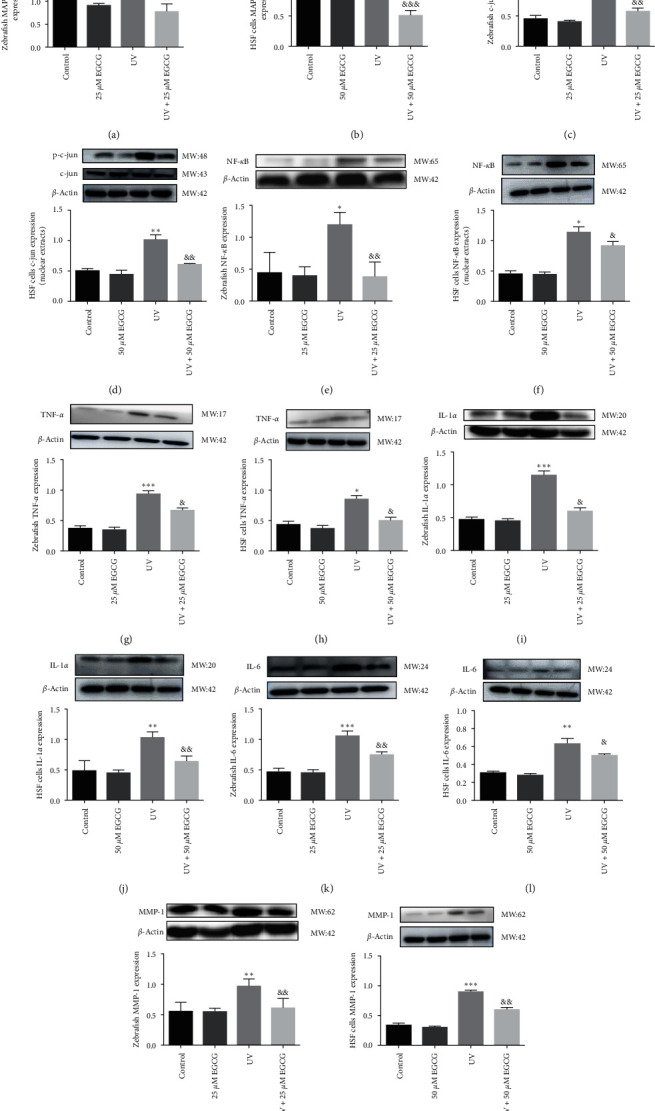
The effects of EGCG on involving photoaging protein expressions. The effects of EGCG on phosphorylation p38 MAPK (a, b), AP-1 (c, d), NF-*κ*B (e, f), TNF-*α* (g, h), IL-1*α* (i, j), IL-6 (k, l), and MMP-1 (m, n) expressions in UVR-induced zebrafish larvae (25 *μ*M EGCG) and HSF cells (50 *μ*M EGCG) for 72 hr. The protein bands of *c*-jun and NF-*κ*B were nuclear extracts of HSFs. The experiments were repeated three times. Data are presented as means ± SD (*n* = 3).  ^*∗*^*P* < 0.05,  ^*∗∗*^*P* < 0.01, and  ^*∗∗∗*^*P* < 0.001 compared with control group. ^&^*P* < 0.05, ^&&^*P* < 0.01, and ^&&&^*P* < 0.001 compared with UVR group.

To investigate the mechanism and effects of EGCG in the suppression of UVR-induced photoaging proteins, including p38 MAPK, *c*-jun, NF-*κ*B, TNF-*α*, IL-1*α*, IL-6, and MMP-1. Our recent results corroborated that EGCG treatment significant inhibition of UVR stressed phosphorylation p38 MAPK (Figures 5(a) and 5(b)), phosphorylation *c*-jun (Figures 5(c) and 5(d)) expressions to fight against skin aging. Moreover, EGCG manifested that marked decrease involved in photoaging related inflammatory factors NF-*κ*B (Figures 5(e) and 5(f)), TNF-*α* (Figures 5(g) and 5(h)), IL-1*α* (Figures 5(i) and 5(j)), and IL-6 (Figures 5(k) and 5(l)) expressions, and obviously downregulated MMP-1 (Figures 5(m) and 5(n)) expression, prevent degrading essentially all components of the ECM.

## 4. Discussion

The overexposure of normal skin to solar UVR may give rise to short-term reactions such as erythema and atypical pigmentation, and long-term responses increasing a risk factor of developing photoaging and skin disorders [20]. Chronological aging in human skin, like photoaging is a cumulative biological process, which leads to the evolutionary modification of structural and functional features [21]. UVR activates a complex biochemical signaling cascade involving oxidative stress and inflammatory response [22]. The free radical overproduction caused by long-term exposure to ultraviolet irradiation thereby disrupting intracellular redox balance, which the interaction between oxidation that the production of free radical and antioxidants that eradicate free radical. When this balance is broken, triggering the release of inflammatory factors, numerous cellular structures and functional, macromolecules, and coenzyme may be alterations and damage, further resulting in the photoaging and skin disorders [5, 23].

It is an effective method to prohibit symptoms related to the skin diseases, especially photoaging with antioxidants [8]. The essential measures to retard or at least minimize free radical-induced photoaging and intrinsic skin aging include fight against UVR and antioxidant homeostasis [4, 24]. It has been reported that green tea and its main components EGCG have antioxidative, anti-inflammatory, and antiaging [19, 25, 26]. EGCG exhibits antioxidant properties to prevent the production of reactive oxygen intermediates, which are produced in the UVR stress photodamages. The study revealed that the potential mechanism of EGCG in protecting against UVA damage is closely related to its potent antioxidant effects and the ability to regulate genes expressions in human fibroblasts or retinal pigment epithelium (RPE) cells [10, 27]. EGCG as potential natural chemopreventive agents during exposure to UVB-induced DNA damage in keratinocytes [28]. However, these studies have mainly concentrated in UVA or UVB-irradiated skin disorders *in vitro* at present. The protective effects of EGCG and how these responses are regulated by UVA + UVB-induced vertebrates photoaging with EGCGs are not well-elucidated.

Zebrafish (*D. rerio*) as the emerging model vertebrates, and 70% of human genes have at least one zebrafish ortholog. The National Institutes of Health (NIH) in the United States ranks zebrafish as the third largest vertebrate model organism after mice and rats. The main advantages of zebrafish as model organisms due to their easy rearing, short life, and experimental cycle (organs can be formed within 24 hr), strong reproductive ability and low cost [29]. The fibroblasts produce a large amount of MMPs after UVR penetrating the skin, which damaged the collagen and elastic fibers, and inhibited collagen synthesis in the dermis [3, 30]. Thereupon, we established the zebrafish and HSF cells photoaging model induced by 10 J/cm^2^ UVA + 30 mJ/cm^2^ UVB irradiation in our study, which lead to a significant degradation of collagen and ECM that determine the skin elasticity. The suppressive effects of 25 or 50 *μ*M EGCG intervention on caudal fin damage, collage reduction, and the SA*β*-gal activities induced by UVR for 72 hr explain why EGCG treatment relieved collagen damage those results in photoaging in zebrafish and HSF cells.

The studies reported that UVA or UVB irradiation increases the production of oxidative free radical and several proinflammatory cytokines [5, 6]. Findings from our study verified that overexposure to UVR increases the production of oxygen radicals, which induces oxidative stress and chronic inflammation reactions. Subsequently, our results indicated that antioxidants EGCG significantly protected zebrafish and cells against UVR-induced oxidative and inflammatory indicators, such as ROS, MDA, 8-OHdG, TNF-*α*, IL-1*α*, and IL-6, and ameliorated the antioxidant enzyme: SOD activity. The above-mentioned findings were consistent with the other studies [4, 25, 31].

p38 MAPK, as a common pathway of intracellular signaling transduction for nuclear reactions after stress stimulation (UVR or H_2_O_2_) and inflammatory factors, which is closely associated with the inflammatory response, cellular proliferation, growth, differentiation, development, and apoptosis [32]. MAPKs activity increases and regulates both the abundance and transactivating capacities of activator protein-1 (AP-1) members. AP-1 is a nuclear transcription factor that activates the transcriptional activity of target genes and regulates cellular proliferation, differentiation, and apoptosis [33]. In response to the external stimuli, AP-1 can be activated by the upstream p38 MAPK signaling pathway through increasing AP-1 synthesis or directly inducing AP-1 activity [34]. C-jun is a member of the Jun family containing *c*-jun, junB, and junD, and is a key component of the transcription factor AP-1. Activated *c*-jun is key regulators of the biological processes such as cell proliferation and apoptosis [35]. Park et al. [36] reported that the photoprotective effect on human skin fibroblasts via MAPK/AP-1/MMP signaling pathway.

Another important nuclear transcription factor NF-*κ*B, UVR-induced oxygen radicals, and proinflammatory cytokines mediated by NF-*κ*B reportedly play critical roles in photoaging and the progression of tumors [37]. The activation of the transcription factor NF-*κ*B involving cell differentiation, apoptosis, inflammatory responses, and p38 MAPK/AP-1 signaling and ultimately leads to the production of proinflammatory mediators, further triggers an increase of MMP-1 activity that degrade collagen and ECM, causing skin photoaging [38]. Bae et al. [39] showed that EGCG has abilities to hamper UVB-induced collagenolytic MMP production via interfering with the MAPK-responsive pathways. Herein, our results demonstrated that activation of p38 MAPK phosphorylation by EGCG to zebrafish and HSF cells and effectively inhibited the activity of the transcriptional factor NF-*κ*B, thereby reducing the *c*-jun and inflammatory factors: TNF-*α*, IL-1*α*, and IL-6 levels. Furthermore, we indicated that MMP1 activities markedly downregulated after EGCG against UVR induced-skin aging.

## 5. Conclusion

Taken together, the results of this study successfully confirm that EGCG ameliorates skin aging via protecting against the oxidative and inflammatory damage caused by UVR-induced photoaging. Additionally, the results further clearly authenticate that the regulation of the p38 MAPK signal pathway is capable of modulating NF-*κ*B, *c*-jun, and MMPs activity after ultraviolet irradiation. To sum up, our study possibly provides critical insights into the EGCG-mediated protective effects on the photodamage in zebrafish and HSF cells.

## Data Availability

The data that support the findings of this study are available from the corresponding author upon reasonable request.
